# C1orf74 positively regulates the EGFR/AKT/mTORC1 signaling in lung adenocarcinoma cells

**DOI:** 10.7717/peerj.13908

**Published:** 2022-08-22

**Authors:** Jinyong Guo, Aili Li, Ruolin Guo, Qiufeng He, Youru Wu, Yi Gou, Junfei Jin, Guojin Huang

**Affiliations:** 1 Laboratory of Respiratory Diseases, The Affiliated Hospital of Guilin Medical University, Guilin, Guangxi, P.R. China; 2 Molecular Medicine in Liver Injury and Repair, The Affiliated Hospital of Guilin Medical University, Guilin, Guangxi, China; 3 Guangxi Health Commission Key Laboratory of Basic Research in Sphingolipid Metabolism Related Diseases, The Affiliated Hospital of Guilin Medical University, Guilin, Guangxi, China; 4 China-USA Lipids in Health and Disease Research Center, Guilin Medical University, Guilin, Guangxi, China

**Keywords:** C1orf74, Lung adenocarcinoma, EGFR/AKT/mTORC1 signaling, A549, H1993, HCC827

## Abstract

**Background:**

Lung adenocarcinoma (LUAD) is a major type of lung cancer with poor prognosis and low 5-year survival rate, which urgently needs further investigation in order to elucidate its mechanisms completely and discover novel therapeutic targets. C1orf74 is a novel protein with unknown function either in normal cells or cancer cells. The aim of this study is to investigate the expression and function of C1orf74 in LUAD cells.

**Methods:**

The expression of C1orf74 in LUAD was analyzed using the LUAD datasets from public databases. The prognostic value of C1orf74 in LUAD was analyzed using Kaplan-Meier Plotter. C1orf74 expression in LUAD cell line A549, H1993 and HCC827 was silenced using small interfering RNA, and then the effects of C1orf74 knockdown on proliferation, migration and invasion of LUAD cells were detected by colony formation assay and Transwell assay, the role of C1orf74 in EGFR/AKT/mTORC1 signaling pathway was examined by Western blot, and the function of C1orf74 in cell cycle was detected by flow cytometry.

**Results:**

The results of LUAD clinical data showed that C1orf74 was upregulated in LUAD tissues, and its high expression was associated with poor prognosis. The results from cultured LUAD cells demonstrated that C1orf74 knockdown inhibited cell proliferation, migration and invasion, but induced cell cycle arrest and autophagy. Moreover, C1orf74 knockdown suppressed EGFR/AKT/mTORC1 signaling in LUAD cells. In conclusion, the present study revealed that C1orf74 is upregulated in LUAD tissues and plays an oncogenic role in LUAD, and that C1orf74 positively regulates cell proliferation and mobility through the EGFR/AKT/mTORC1 signaling pathway in LUAD cells.

## Introduction

Lung cancer is the main cause of cancer death worldwide, accounting for about 18% of all cancer deaths (Sung et al., 2021). There are two main histological types of lung cancer: small cell carcinoma and non-small cell carcinoma (NSCLC), which includes adenocarcinoma, squamous cell carcinoma, small cell carcinoma, and large cell carcinoma (Herbst, Morgensztern & Boshoff, 2018). NSCLC accounts for about 85% lung cancer cases, and lung adenocarcinoma (LUAD) accounts for about 40% of NSCLC cases (Herbst, Morgensztern & Boshoff, 2018). Although current treatments are of great help to patients with lung cancer, the prognosis of patients with lung cancer is still poor, with a 5-year survival rate of only about 10% to 20% in most countries (Sung et al., 2021). Due to the high degree of malignancy, rapid disease progression, poor prognosis and high mortality, it is necessary to further improve our understanding of the pathogenesis of lung cancer and discover new therapeutic targets.

Cell proliferation is tightly regulated to maintain tissue homeostasis under normal circumstances (Hanahan & Weinberg, 2011). However, in cancer cells, certain mutations and/or epigenetic changes result in continuous and even enhanced proliferation signals, which lead to excessive cell proliferation and thus contribute to tumorigenesis (Hanahan & Weinberg, 2011). The epidermal growth factor receptor (EGFR)/AKT/mechanistic target of rapamycin (mTOR) signaling pathway is one of the most important pathways, which is considered as a master regulator for cancer (Manning & Toker, 2017; Wee & Wang, 2017). AKT is a well-characterized kinase that plays a crucial role in the pathogenesis of many human cancers (Manning & Toker, 2017). The highly conserved protein kinase mTOR is a central cell growth regulator connecting cellular metabolism and growth with a wide range of environmental inputs as a key component of mTOR complex 1 (mTORC1) and mTORC2 (Liu & Sabatini, 2020). As a pivot point between anabolic and catabolic processes, mTORC1 signaling has established roles in regulating metabolism, translation and autophagy (Liu & Sabatini, 2020). mTORC1 regulates protein synthesis and cell growth through its downstream target molecules: 4E-BP1 and S6K (Liu & Sabatini, 2020).

C1orf74 refers to protein encoded by C1orf74 (Chromosome 1 Open Reading Frame 74) gene located at 1q32.2, but its function is still unknown either in normal cells or cancer cells. In the present study, we investigate C1orf74’s function, mechanism and association with prognosis in LUAD.

## Methods and materials

### Antibodies and small interfering (si)RNA

The antibody against C1orf74 (cat. no. GTX120342-S) was purchased from GeneTex, Inc.. Antibodies against p62 (cat. no. 39749S), p70 S6 kinase (S6K; cat. no. 9202), p‑S6K (cat. no. 9204), AKT (cat. no. 9272S), p-AKT (cat. no. 4060S), cyclinD1 (cat. no. 60186-1-lg) and HRP‑conjugated anti‑mouse (cat. no. 7076) and anti‑rabbit (cat. no. 7074) IgG antibodies were purchased from Cell Signaling Technology, Inc.. The antibody against microtubule‑associated proteins 1A/1B light chain 3B (LC3B; cat. no. ab192890) was obtained from Abcam, and the antibody against GAPDH (cat. no. TA505454) was purchased from OriGene Technologies, Inc. The dilutions of the antibodies used for western blotting were 1:500 for C1orf74, 1:800 for LC3B, AKT, S6K, 1:1,000 for p62, p‑AKT and p‑S6K, 1:2,000 for cyclinD1 and 1:5,000 for HRP‑conjugated anti‑mouse and anti‑rabbit IgG antibodies.

C1orf74 siRNA (C1orf74 siRNA-1, 5′-GCCAGCUGUGCUCUAUGAUTT-3′; C1orf74 siRNA-2, 5′- GCUUGGUACCAUAGCCUUUTT -3′; C1orf74 siRNA -3′, 5′- CCUCUCAGACUGGAAUTT -3′) and the negative control siRNA (control siRNA, 5′‑UUC UCC GAA CGU GUC ACG UTT‑3′) were synthesized by Shanghai GenePharma Co. Ltd (Shanghai, China).

### Cell culture and transfection

The A549 cell line was from Kunming Cell Bank, Chinese Academy of Sciences (Kunming, Yunnan, China). HCC827 and NCI-H1993 were obtained from Meisen CTCC (Meisen Cell Biotechnology Co., Ltd, Zhejiang, Hangzhou, China). Cells were cultured in RPMI-1640 medium (Gibco, Thermo Fisher Scientific, Inc., Waltham, MA, USA) with 10% fetal bovine serum (Gibco, Thermo Fisher Scientific, Inc., Waltham, MA, USA) at 37 °C in a 5% CO_2_ incubator. The cells were seeded into 12-well plates (0.9 × 10^5^ cells/well) and cultured overnight to 60–70% confluence, and then transfected with C1orf74 siRNA (100 pmol) or control siRNA(100 pmol) using Lipofectamine 3000 (Invitrogen, Carlsbad, CA, USA) according to the manufacturer’s instruction.

### Colony formation assay

The assay was performed as described previously (Wang et al., 2021). In brief, at 48 h post-transfection, cells were seeded into six-well plate in 2 ml growth medium at a density of 0.6 × 10^3^ and cultured in RPMI-1640 with 10% FBS at 37 °C in a humidified incubator with 5% CO_2_ for 10–15 days. The culture medium was changed every 2 days. Following incubation, cells were stained with 2 ml of 1% crystal violet solution. Finally, the plates were dried and scanned with Epson Perfection V370 Photo scanner. Cell colonies were counted manually.

### Invasion and migration assays

These assays were conducted as previously described (Han et al., 2021). At 24 h post-transfection, 3 × 10^3^ cells in FBS‑free medium were seeded into a 24‑well Transwell insert (8‑µm pore size; BD Biosciences, Franklin Lakes, NJ, USA) with or without Matrigel®. Medium supplemented with 20% FBS was added to the lower chamber. After incubation at 37 °C with 5% CO_2_ for 24 h, non‑invading or non‑migrating cells were removed from the top wells with a cotton swab. The cells that had transgressed to the bottom of the membrane were fixed with 4% paraformaldehyde at room temperature for 15 min and stained with 0.2% crystal violet at 4 °C overnight. Images of five randomly selected independent fields from each well were captured under an Olympus TH4‑200 phase‑contrast microscope (×200 magnification; Olympus Corporation, Shinjuku City, Tokyo, Japan). The cells in each image were quantified with ImageJ 1.53a software, and the results were presented as a percentage of the control group.

### Cell cycle assay

Transfected cells were collected and washed twice with ice cold phosphate buffer saline (PBS; Gibco Invitrogen, Waltham, MA, USA). The cells were then fixed with 70% ethanol overnight at 4 °C. On the day of analysis, the cells were washed with ice cold PBS and then stained with the staining solution [0.2 mg RNase A+PI/TritonX-100 (20 µg PI +0.1%TritonX-100)] in dark for 15 min at 37 °C. Finally, the cell cycle was analyzed by flow cytometry (NovoCyte 2060R; ACEA Biosciences, Inc., San Diego, CA, USA) with Software NovoExpress 1.4.0 (ACEA Biosciences, Inc., San Diego, CA, USA).

### Western blot analysis

Western blot analysis was conducted as described previously (Du et al., 2021). Briefly, the cells were washed three times with ice cold PBS, and lysed with RIPA buffer containing 1% protease and 1% phosphatase inhibitor (Boston Bioproducts, Milford, MA, USA). Protein concentration of cell lysates was determined using BCA kit (Beyotime Institute of Biotechnology, Shanghai, China). 30 µg lysate was resolved by SDS-PAGE and transferred onto PVDF membrane. Subsequently, the membrane was incubated with the primary antibodies at 4 °C overnight, washed with 1X TBST buffer, and incubated with the secondary antibody (HRP‑conjugated anti‑mouse or anti‑rabbit IgG antibody) at room temperature for 1 h. Blots were developed using the SuperSignal™ West Femto substrate (Bridgen Biotechnology Co., Ltd., Beijing, China), and visualized using Amersham ImageQuant 800 imaging system (Cytiva, Marlborough, MA, USA) or x-ray films. The optical density of the protein bands was semi‑quantified with ImageJ 1.53a software (National Institutes of Health) and normalized to that of the loading control.

### Gene set enrichment analysis

TCGA LUAD gene expression dataset was downloaded through the Xena browser (https://xenabrowser.net/). Tumor samples in TCGA LUAD dataset were classified into high- and low-C1orf74 groups using median value of C1orf74 expression as cut-off. Then gene set enrichment was analyzed using GSEA 4.0.3 software (downloaded from http://www.broad.mit.edu/gsea/) with the pre-defined hallmark gene sets. Permutation number was set as 1,000. A gene set is considered significantly enriched when the false discovery rate (FDR) score <0.25.

### Analysis of C1orf74 expression in LUAD tissues and its prognostic values

The analysis was performed as described previously (Wang et al., 2020). In brief, the C1orf74 expression in LUAD tissues of The Cancer Genome Atlas (TCGA) LUAD was analyzed using online tool UALCAN (http://ualcan.path.uab.edu/) (Chandrashekar et al., 2017), and in LUAD tissues of GSE75037 and GSE31210 was analyzed using GraphPad Prism 8.0. The prognostic values of C1orf74 in patients with LUAD were analyzed using online software Kaplan-Meier Plotter (http://kmplot.com) (Győrffy et al., 2013).

### Statistical analysis

Statistical analysis was performed using GraphPad Prism 8 software (GraphPad Software, Inc., San Diego, CA, USA). All experiments were conducted independently at least three times, and the data were expressed as mean ± standard deviation. Student’s t test and ANOVA test were used for analysis. *P* < 0.05 was considered statistically significant.

## Result

### The expression of C1orf74 was upregulated in LUAD tissues, and its high expression was associated with unfavorable prognosis of patients with LUAD

**Figure 1:**
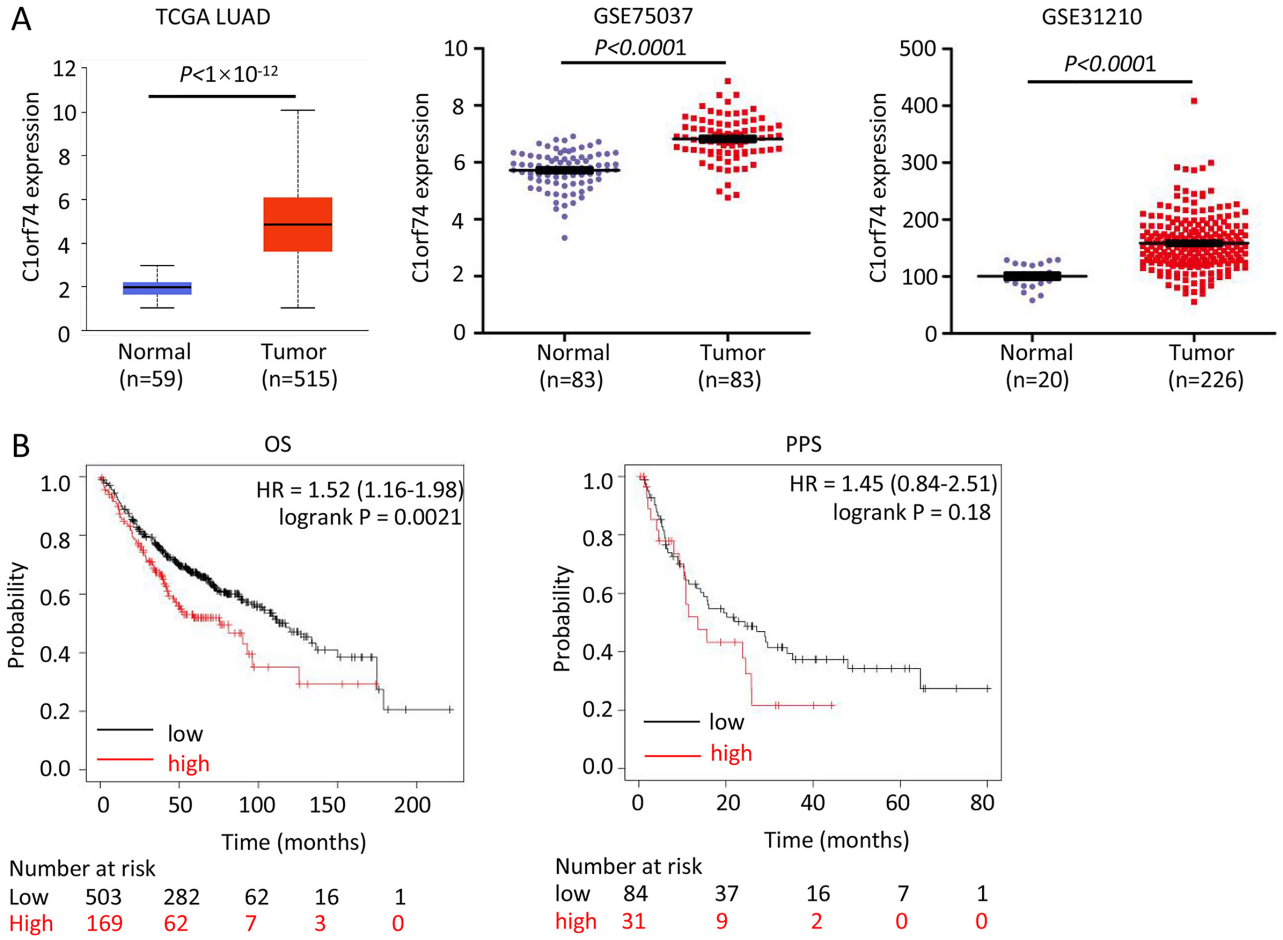
C1orf74 is upregulated in LUAD and associated with unfavorable prognosis. (A) C1orf74 expression levels in LUAD and normal lung tissues from TCGA LUAD data, GSE75037 and GSE31210 were analyzed using UALCAN or Prism 8. MYG1 expression levels were significantly higher in LUAD compared with those in normal tissues. (B) High expression levels of C1orf74 were associated with unfavorable OS (cut-off value, 163; HR = 1.52; 95% CI [1.16–1.98]; log-rank P = 0.0021), but was not associated with PPS (cut-off value, 166; HR = 1.45; 95% CI [0.84–2.51]; log-rank P = 0.18). Patients were separated using an auto-select best cutoff. OS, overall survival; PPS, post-progression survival. LUAD, lung adenocarcinoma; TCGA, The Cancer Genome Atlas.

To reveal C1orf74’s role, we firstly analyzed its expression in LUAD and association with prognosis of patients with LUAD. We analyzed its expression in three LUAD datasets-TCGA LUAD, GSE75037 and GSE31210, and found that the expression of C1orf74 in LUAD tissues was significantly upregulated in all three LUAD datasets (Fig. 1A). In order to further clarify the relationship between the level of C1orf74 expression and the prognosis of patients with LUAD, Kaplan-Meier plotter was used to analyze the prognosis of patients with LUAD in the database (http://kmplot.com/). The results showed that patients with high expression of C1orf74 had a significantly lower overall survival (OS) rate and post progression survival rate (PPS) than patients with low expression of C1orf74 [OS: Log rank P = 0.0017, HR = 1.3 (1.1–1.53); PPS: Log rank P = 0.023, HR = 1.7 (1.07–2.68)] (Fig. 1B), indicating that C1orf74 was positively associated with LUAD progression.

### C1orf74 promotes the proliferation of LUAD cells

**Figure 2:**
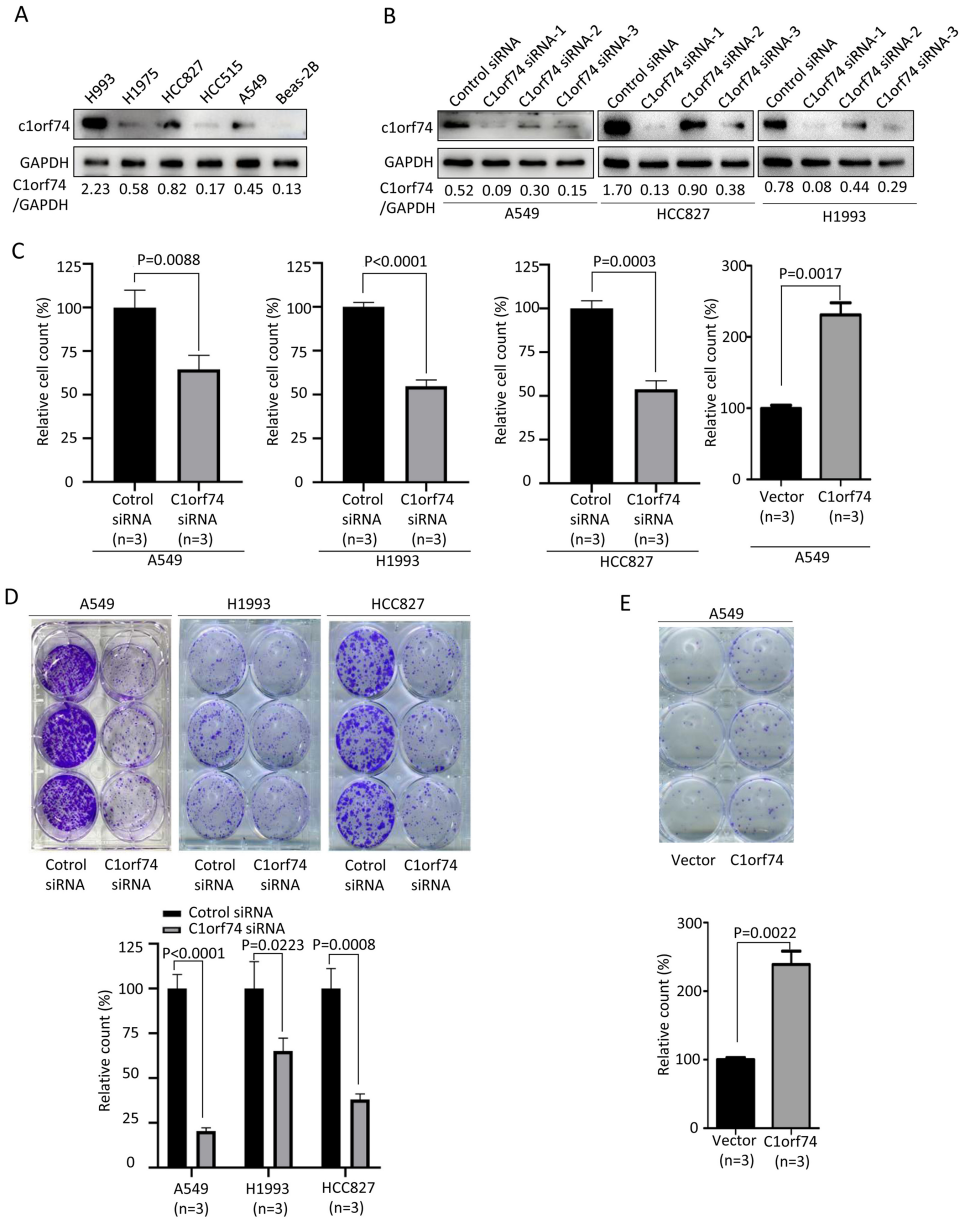
C1orf74 promotes cell proliferation and colony formation of lung adenocarcinoma cells. (A) Western blot results of C1orf74 expression in a panel of LUAD and normal lung cells. (B) The effects of C1orf74 siRNA on its protein expression in A549, HCC827 and H1993 cells. (C) C1orf74 promotes cell proliferation. A549, H1993 and HCC827 cells were transfected with C1orf74 siRNA and control siRNA for 48 h , A549 cells were transfected with C1orf74 plasmids and empty vectors for 48 h. Cell proliferation was determined by counting the number of cells. (D) C1orf74 knockdown inhibits colony formation in A549 , H1993 and HCC827 cells. (E) C1orf74 overexpression promotes colony formation in A549. siRNA, small interfering RNA.

To understand how C1orf74 to affect LUAD progression, we performed *in vitro* experiments to investigate its effects on some of the hallmarks of cancer cells (Hanahan & Weinberg, 2011). We firstly examined C1orf74 expression in a panel of LUAD cell lines (A549, HCC827, HCC515, H1975 and H1993) and normal lung cell line BEAS-2B by Western blot to identify cell lines for our experiments. The results showed that C1orf74 was highly expressed in H993 cells, while weakly expressed in HCC827 and A549 cells (Fig. 2A). Therefore, A549, H1993 and HCC827 were selected to conduct subsequent experiments. In order to down-regulate C1orf74 expression, three C1orf74 siRNAs (C1orf74 siRNA-1–3) were synthesized and transfected into LUAD cells, respectively, and then their inhibitory effects on C1orf74 expression were determined by Western blot at 48 h after transfection. The results showed that C1orf74 siRNA-1 had the strongest inhibitory effect in all three cell lines (Fig. 2B). Therefore, C1orf74 siRNA-1 was used for the experiments followed.

Excessive cell proliferation is a typical feature of cancer cells (Hanahan & Weinberg, 2011). To assess C1orf74’s potential role on cell proliferation, A549, H1993 and HCC827 were transfected with C1orf74 siRNA or control siRNA for 48 h, and A549 cells were transfected with C1orf74 plasmids or empty vectors for 48 h, respectively, and then cell numbers were counted. The results demonstrated that the cell numbers of C1orf74 siRNA groups were decreased significantly compared with control groups (Fig. 2C), and C1orf74 overexpression group was increased remarkably compared with empty vector group (Fig. 2C). Furthermore, colony formation assay was utilized to assess the ability of a single cell to grow into colony upon C1orf74 knockdown and overexpression. The results indicated that the colony numbers of C1orf74 siRNA groups were reduced significantly compared with those of control groups in all three cell lines (Fig. 2D), and the colony numbers of C1orf74 overexpression group was increased significantly compared with those of empty vector group in A549 cell lines (Fig. 2E). These data suggested that C1orf74 promotes cell proliferation.

### C1orf74 knockdown induces cell cycle arrest in LUAD cells

**Figure 3:**
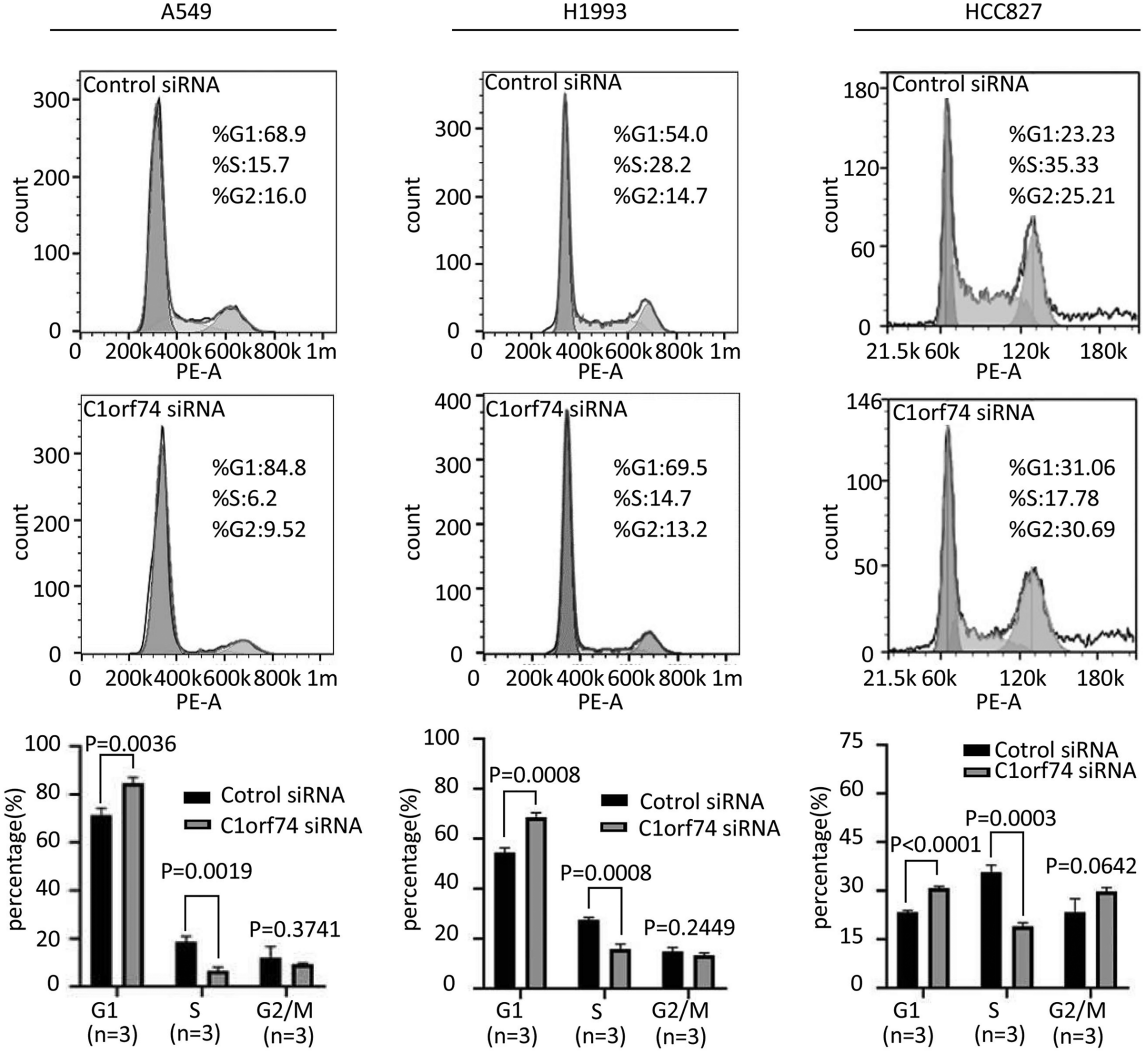
C1orf74 knockdown induces cell cycle arrest in lung adenocarcinoma cells. A549, H1993 and HCC827 cells were transfected with C1orf74 siRNA for 48 h, and cell cycle was determined by flow cytometry. The results demonstrated that C1orf74 knockdown led to the increase of the proportion at G1 in A549, H1993 and HCC827 cells. siRNA, small interfering RNA.

To uncover how C1orf74 is involved in the regulation of cell proliferation, we analyzed cell cycle upon C1orf74 knockdown. A549, H1993 and HCC827 cells were transfected with C1orf74 siRNA or control siRNA for 48 h, respectively, and then the cell cycle was analyzed using cell flow cytometry. The results indicated that the proportion of the cells in G1 phase was significantly increased, whereas the proportion of the cells in S phase was significantly decreased upon C1orf74 knockdown in all three cell lines (Fig. 3), suggesting that C1orf74 can promote cell cycle.

### C1orf74 enhances migration and invasion of LUAD cells

**Figure 4:**
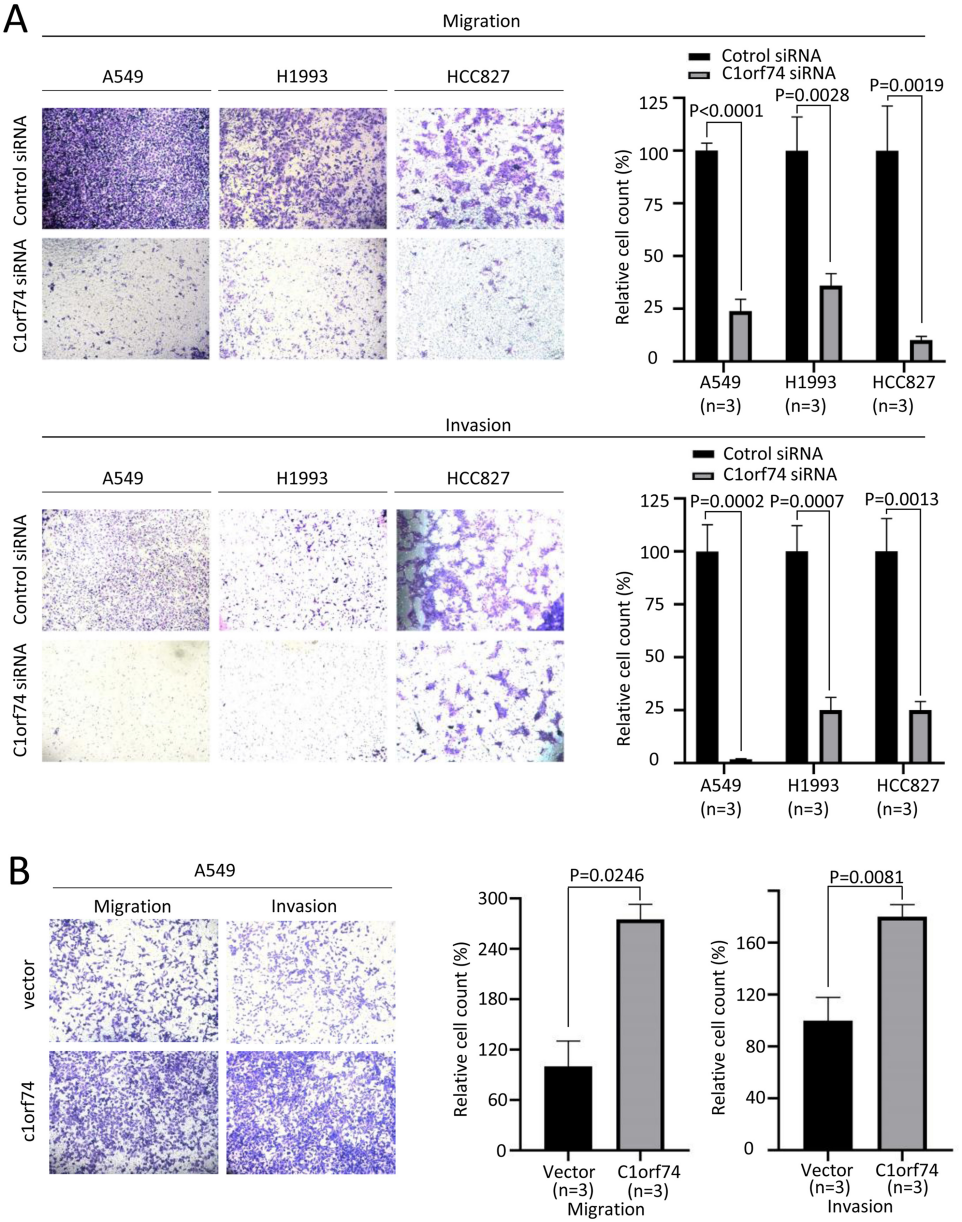
C1orf74 promotes the migration and invasion of lung adenocarcinoma cells. The results of the Transwell assays demonstrated that (A) C1orf74 knockdown impaired the migration and invasion of A549 and H1993 cells, and (B) C1orf74 overexpression enhanced the migration and invasion of A549. siRNA, small interfering RNA.

Increased cellular capabilities of migration and invasion promote tumor local invasion and distant metastasis, which is connected to worse prognosis (Hanahan & Weinberg, 2011). Therefore, A549, H1993 and HCC827 cells were transfected with C1orf74 siRNA or control siRNA for 48 h, and A549 cells were transfected with C1orf74 plasmids or empty vectors for 48 h, and then the migration and invasion of the cells were analyzed using Transwell (with or without gel) assay. The results demonstrated that, compared with the control, C1orf74 knockdown led to the decreased rates of migration and invasion in all three cell lines (Fig. 4A), and C1orf74 overexpression enhanced migration and invasion in A549 cell lines (Fig. 4B), indicating that C1orf74 promotes cell migration and invasion.

### C1orf74 knockdown suppresses AKT/mTORC1 signaling and cyclin D 1 expression in LUAD cells

**Figure 5:**
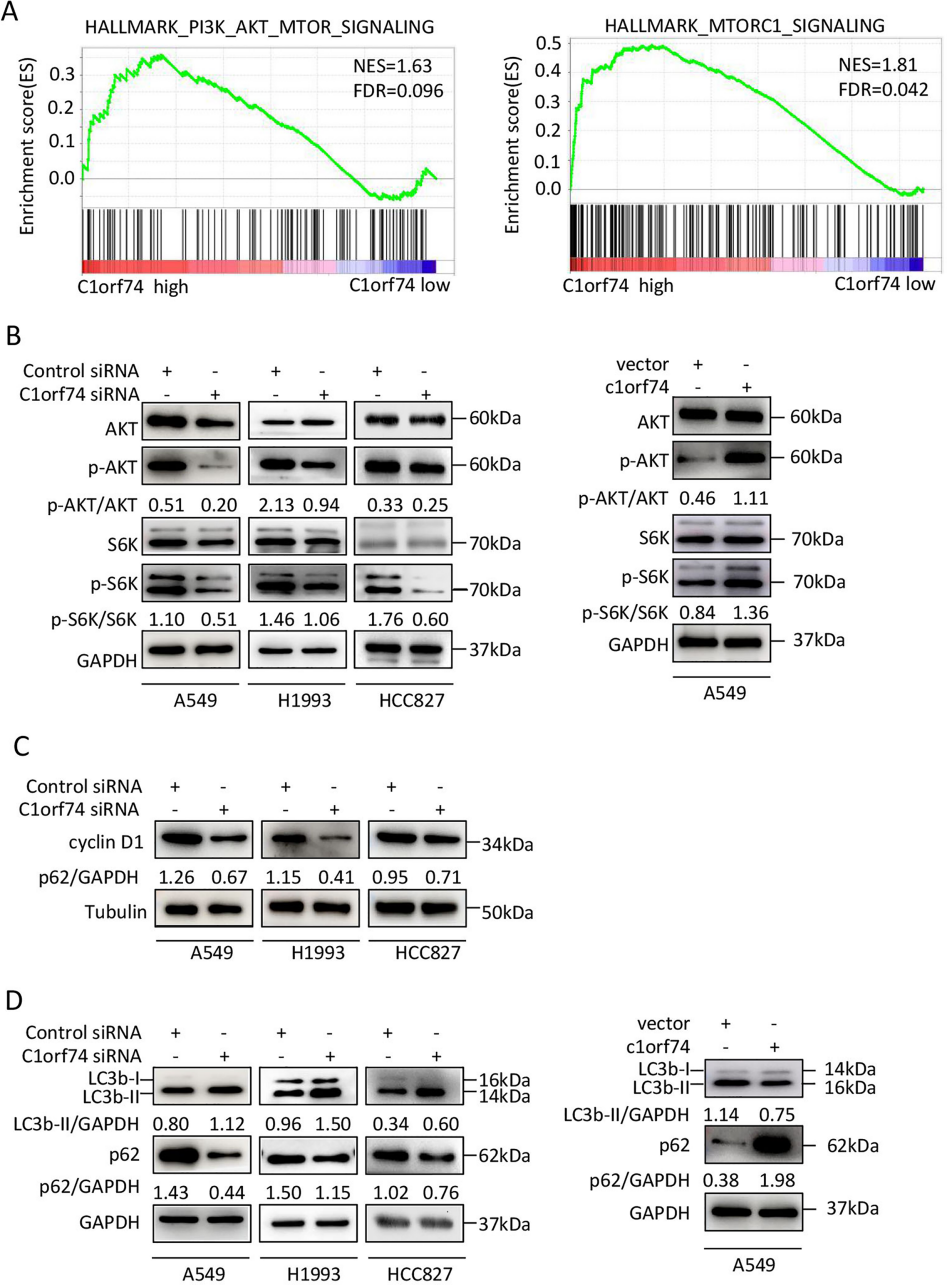
C1orf74 positively regulates the AKT/mTORC1 signaling pathway. (A) The Gene Set Enrichment Analysis results indicated that the gene sets of ‘HALLMARK_PI3K_AKT_MTOR_SIGNALING’ and ‘HALLMARK_MTORC1_SIGNALING’ were enriched with high expression of C1orf74. (B) Knockdown of C1orf74 inhibited the levels of phosphorylation of AKT and p70S6K compared with those in the control siRNAtransfected group. Overexpression of C1orf74 enhanced the phosphorylation of AKT and p70S6K. (C) Knockdown of C1orf74 inhibited the expression of cyclin D1; (D) Knockdown of C1orf74 enhanced the conversion of LC3-I to LC3-II and the degradation of p62 compared with those in the control siRNA group, meanwhile overexpression of C1orf74 had opposite effects on autophagy. NES, normalized enrichment score; FDR, false discovery rate; siRNA, small interfering RNA; mTORC1, mTOR complex 1; p-, phosphorylated.

To elucidate the mechanisms underlying C1orf74, the TCGA LUAD gene expression data were classified into C1orf74 high and C1orf74 low group by using median value of C1orf74 gene expression, and then were analyzed using GSEA software to find the potential connections between C1orf74 expression and cell signaling pathways. The result indicated that the gene signatures of PI3K/AKT/mTOR signaling pathway and mTORC1 signaling pathway were highly enriched with C1orf74 high expression (Fig. 5A).

To verify the bioinformatical findings in cultured cells, A549, HCC1993 and HCC827 cells were transfected with C1orf74 siRNA or control siRNA for 48 h, respectively, and the protein levels of p-S6K, S6K, p-AKT and AKT were detected by Western blot. The results showed that the C1orf74 knockdown resulted in the decreased protein levels of p-S6K and p-AKT, but did not affect the total protein levels of S6K and AKT (Fig. 5B). Whereas overexpression of C1orf74 in A549 cell had opposite effects on the protein levels of p-S6K and p-AKT, but did not affect the total protein levels of S6K and AKT (Fig. 5B). These data indicated that C1orf74 plays a positive role in the AKT/mTORC1 signaling.

As we observed that C1orf74 knockdown induced cell cycle arrest in the above experiments, and mTORC1 positively regulates cyclin D1 expression, a key protein of cell cycle (Averous, Fonseca & Proud, 2008), we hypothesized that cyclin D1 expression might be altered upon C1orf74 knockdown. To test this, A549, H1993 and HCC827 cells were transfected with C1orf74 siRNA for 48 h, and then the cyclin D1 was detected by Western blot. The results showed that cyclin D1 was indeed decreased upon C1orf74 knockdown (Fig. 5C).

### C1orf74 suppresses autophagy

Autophagy is a cellular recycle process that acts as tumor suppressive mechanism during tumor initiation period (Mizushima & Levine, 2020), which is negatively regulated by mTORC1 (Liu & Sabatini, 2020). As mTORC1 activity was decreased upon C1orf74 knockdown, we thus examined whether autophagy was affected under this circumstance. A549, H1993 and HCC827 cells were transfected with C1orf74 siRNA or control siRNA for 48 h, and then the autophagic markers LC3 and p62/SQSTM1 protein were detected by Western blot. The results showed that C1orf74 knockdown promoted the conversion of LC3-I to LC3-II and the degradation of P62/SQSTM1 protein (Fig. 5D), meanwhile overexpression of C1orf74 had opposite effects on the protein levels of LC3 and p62/SQSTM1 (Fig. 5D). These results suggested that C1orf74 *suppresses* autophagy in LUAD cells.

### C1orf74 enhances epidermal growth factor (EGF)-induced activation of AKT/mTORC1 signaling in LUAD cells

**Figure 6:**
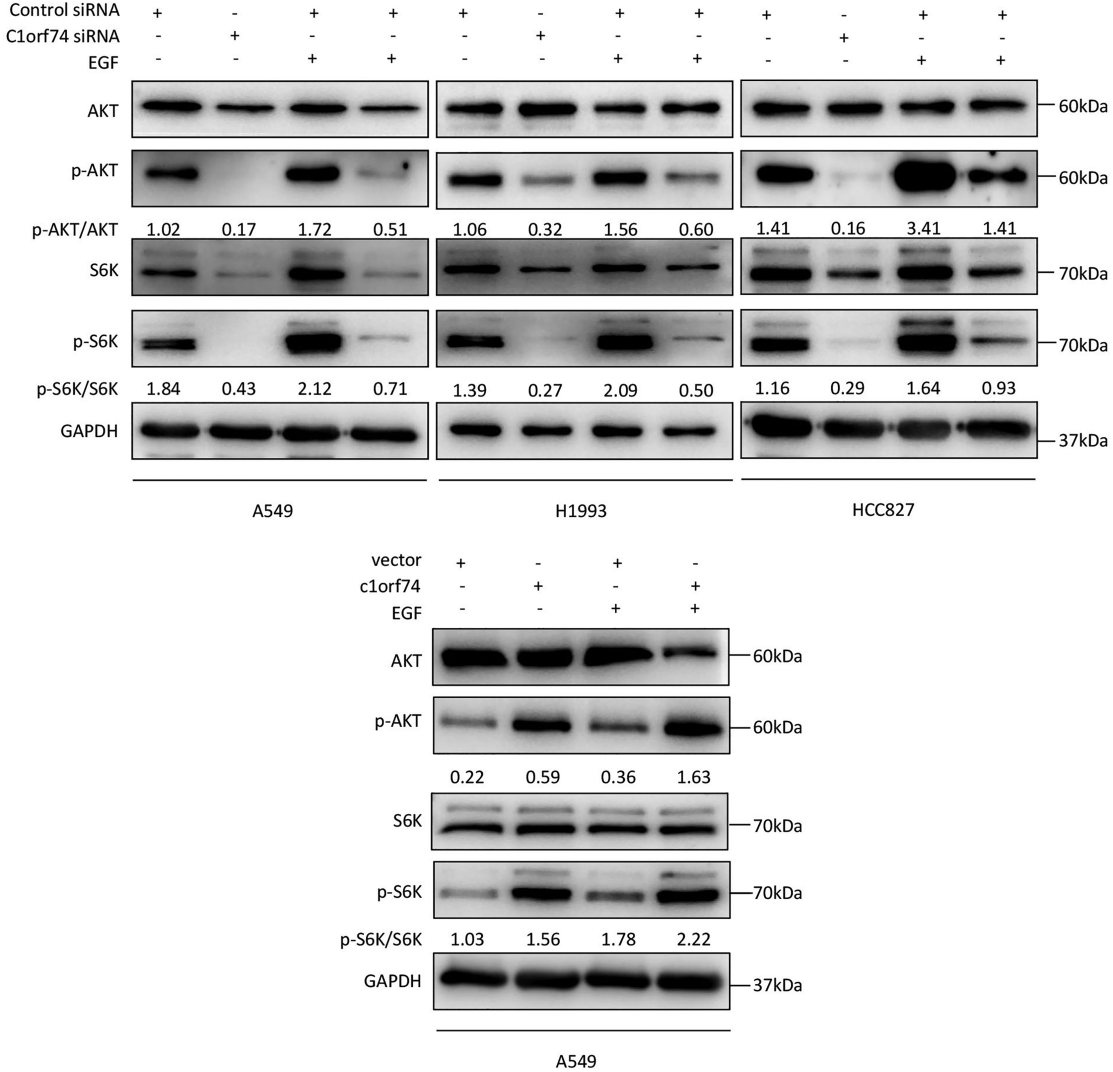
C1orf74 enhances EGF-induced phosphorylation of AKT and p70S6K in lung adenocarcinoma cells. The results of the western blot analysis demonstrated that C1orf74 knockdown suppressed the basal level and EGF-induced phosphorylation of AKT and p70S6K in A549, H1993 and HCC827 cells compared with those in the control siRNA-transfected cells, and that overexpression of C1orf74 enhanced the phosphorylation of AKT and p70S6K. siRNA, small interfering RNA.

As the AKT/mTORC1 signaling is a crucial signaling axis downstream of EGF receptor which has high mutation rate in lung cancers, we asked whether C1orf74 has any role in EGF-stimulated activities of AKT and mTORC1 in LUAD cells. To answer this question, A549, HCC827 and H1993 cells were transfected with C1orf74 siRNA or control siRNA for 48 h, and A549 was transfected with C1orf74 plasmids or control vector for 48 h, followed by EGF (100 ng/mL) treatment for 30 min, and then the phosphorylation of AKT and S6K was determined by Western blot. The results showed that C1orf74 knockdown inhibited EGF-induced phosphorylation of AKT and S6K, while C1orf74 overexpression enhanced EGF-induced phosphorylation of AKT and S6K (Fig. 6). These data suggested that C1orf74 positively regulates EGFR/AKT/mTORC1 signaling.

## Discussion

C1orf74 is a protein localized in plasma membrane and cytosol (Uhlén et al., 2015), but its function and underlying mechanisms still remain unknown in either normal cells or cancer cells. C1orf74 emerged when we mined LUAD prognosis database to discover novel molecules associated with prognosis. In the present report, we observed that C1orf74 was highly expressed in LUAD tumor tissues and its high expression is associated with unfavorable prognosis of patients with LUAD. We demonstrated *in vitro* that C1orf74 knockdown impaired cell proliferation, migration and invasion, and induced cell cycle arrest. Moreover, we revealed that C1orf74 can positively regulate EGFR/AKT/mTORC1 signaling.

As we herein investigated C1orf74 expression only in three LUAD datasets, it is better to study its expression in more datasets to confirm C1orf74’s upregulation in LUAD. Moreover, it is worth to investigate C1orf74’s expression in other types of cancers, so that we can obtain the idea whether C1orf74’s upregulation is specific phenomenon in LUAD or common in most cancers. More importantly, it is necessary to study in the future how C1orf74 expression is upregulated in LUAD, which will not only lets us understand the regulating mechanisms of C1orf74 expression, but also discover upstream molecules that has crucial function in the tumorigenesis of LUAD. In addition, although we observed C1orf74 high expression is associated with unfavorable prognosis, whether C1orf74 can be an independent indicator of prognosis is a question to be addressed.

Excessive cell proliferation as well as enhanced mobility and invasion are cancer hallmarks that contribute to tumor growth and metastasis, and ultimately to unfavorable prognosis of patients (Hanahan & Weinberg, 2011). Here we demonstrated that C1orf74 knockdown repressed cell proliferation, mobility and invasion, which provided cellular evidences to explain how C1orf74 to affect prognosis. Furthermore, we identified C1orf74 as a novel positive regulator of EGFR/AKT/mTORC1 signaling in LUAD cells. As it is well established that the EGFR signaling can promote a multitude of biological processes, including cell proliferation, cell motility and metastasis (Wee & Wang, 2017), our findings not only provides mechanistic explanation about how C1orf74 to affect proliferation as well as cell motility and invasion, but also suggest that C1orf74 could be a potential interfering target to overcome drug resistance against tyrosine kinase inhibitors, which is often observed in patients whose genome has certain EGFR mutations (Herbst, Morgensztern & Boshoff, 2018). To further understand the detail how C1orf74 is involved in EGFR/AKT/mTORC1 signaling, it is essential to identify C1orf74’s interacting protein partners through which it is involved in the regulation of this signaling pathway.

Although the present study, to the best of our knowledge, was the first to reveal C1orf74’s function, underlying mechanism as well as its association with cancer, there are some limitations exist. Because this study used LUAD clinical data and cultured LUAD cells, it is needed to conduct *in vivo* xenograft and metastasis experiments of LUAD cells to further verify our findings. Moreover, studying C1orf74’s role, mechanism and association with prognosis in other types of cancers will provide helpful evidences to completely understand C1orf74’s role in tumorigenesis. Additionally, investigating C1orf74’s function and mechanisms in normal cells and tissue will throw light on C1orf74’s role in physiological context.

## Conclusions

In summary, C1orf74 is highly expressed in LUAD tissues and is associated with poor prognosis. C1orf74 promotes the proliferation, invasion and migration of LUAD cells at least partially through the EGFR/AKT/mTORC1 signaling pathway, and thus it might be a potential therapeutic target for LUAD.

## Supplemental Information

10.7717/peerj.13908/supp-1 Supplemental Information 1 Raw data.

## Abbreviations

LUAD: lung adenocarcinoma
NSCLC: non-small cell carcinoma
EGF: epidermal growth factor
EGFR: epidermal growth factor receptor
mTOR: mechanistic target of rapamycin
mTORC1: mTOR complex 1
LC3: microtubule‑associated proteins 1A/1B light chain 3

## Additional Information and Declarations

### Data Availability

The following information was supplied regarding data availability: The raw data is available in the Supplemental Files.

## References

[ref-1] Averous J, Fonseca BD, Proud CG (2008). Regulation of cyclin D1 expression by mTORC1 signaling requires eukaryotic initiation factor 4E-binding protein 1. Oncogene.

[ref-2] Chandrashekar DS, Bashel B, Balasubramanya SAH, Creighton CJ, Ponce-Rodriguez I, Chakravarthi BVSK, Varambally S (2017). UALCAN: a portal for facilitating tumor subgroup gene expression and survival analyses. Neoplasia.

[ref-3] Du L, Wu Y, Han X, Wang C, Li A, Huang G (2021). NICE‑3‑knockdown induces cell cycle arrest and autophagy in lung adenocarcinoma cells via the AKT/mTORC1 signaling pathway. Experimental and Therapeutic Medicine.

[ref-4] Győrffy B, Surowiak P, Budczies J, Lánczky A (2013). Online survival analysis software to assess the prognostic value of biomarkers using transcriptomic data in non-small-cell lung cancer. PLOS ONE.

[ref-5] Han X, Li A, Wang W, Du L, Wang C, Huang G (2021). MYG1 promotes proliferation and inhibits autophagy in lung adenocarcinoma cells via the AMPK/mTOR complex 1 signaling pathway. Oncology Letters.

[ref-6] Hanahan D, Weinberg RA (2011). Hallmarks of cancer: the next generation. Cell.

[ref-7] Herbst RS, Morgensztern D, Boshoff C (2018). The biology and management of non-small cell lung cancer. Nature.

[ref-8] Liu GY, Sabatini DM (2020). mTOR at the nexus of nutrition, growth, ageing and disease. Nature Reviews Molecular Cell Biology.

[ref-9] Manning BD, Toker A (2017). AKT/PKB signaling: navigating the network. Cell.

[ref-10] Mizushima N, Levine B (2020). Autophagy in human diseases. New England Journal of Medicine.

[ref-11] Sung H, Ferlay J, Siegel RL, Laversanne M, Soerjomataram I, Jemal A, Bray F (2021). Global cancer statistics 2020: GLOBOCAN estimates of incidence and mortality worldwide for 36 cancers in 185 countries. CA: a Cancer Journal for Clinicians.

[ref-12] Uhlén M, Fagerberg L, Hallström BM, Lindskog C, Oksvold P, Mardinoglu A, Sivertsson Å, Kampf C, Sjöstedt E, Asplund A, Olsson I, Edlund K, Lundberg E, Navani S, Al-Khalili Szigyarto C, Odeberg J, Djureinovic D, Takanen JO, Hober S, Alm T, Edqvist P-H, Berling H, Tegel H, Mulder J, Rockberg J, Nilsson P, Schwenk JM, Hamsten M, von Feilitzen K, Forsberg M, Persson L, Johansson F, Zwahlen M, von Heijne G, Nielsen J, Pontén K (2015). Tissue-based map of the human proteome. Science.

[ref-13] Wang W, Li A, Han X, Wang Q, Guo J, Wu Y, Wang C, Huang G (2020). DEPDC1 up‐regulates RAS expression to inhibit autophagy in lung adenocarcinoma cells. Journal of Cellular and Molecular Medicine.

[ref-14] Wang C, Wang W, Han X, Du L, Li A, Huang G (2021). Methyltransferase‑like 1 regulates lung adenocarcinoma A549 cell proliferation and autophagy via the AKT/mTORC1 signaling pathway. Oncology Letters.

[ref-15] Wee P, Wang Z (2017). Epidermal growth factor receptor cell proliferation signaling pathways. Cancers.

