# Supplementary figures and images for: C1orf74 positively regulates the EGFR/AKT/mTORC1 signaling in lung adenocarcinoma cells
Source: PeerJ. 2022 Aug 22;10:e13908. doi: 10.7717/peerj.13908 (PMC9406791; doi:10.7717/peerj.13908)

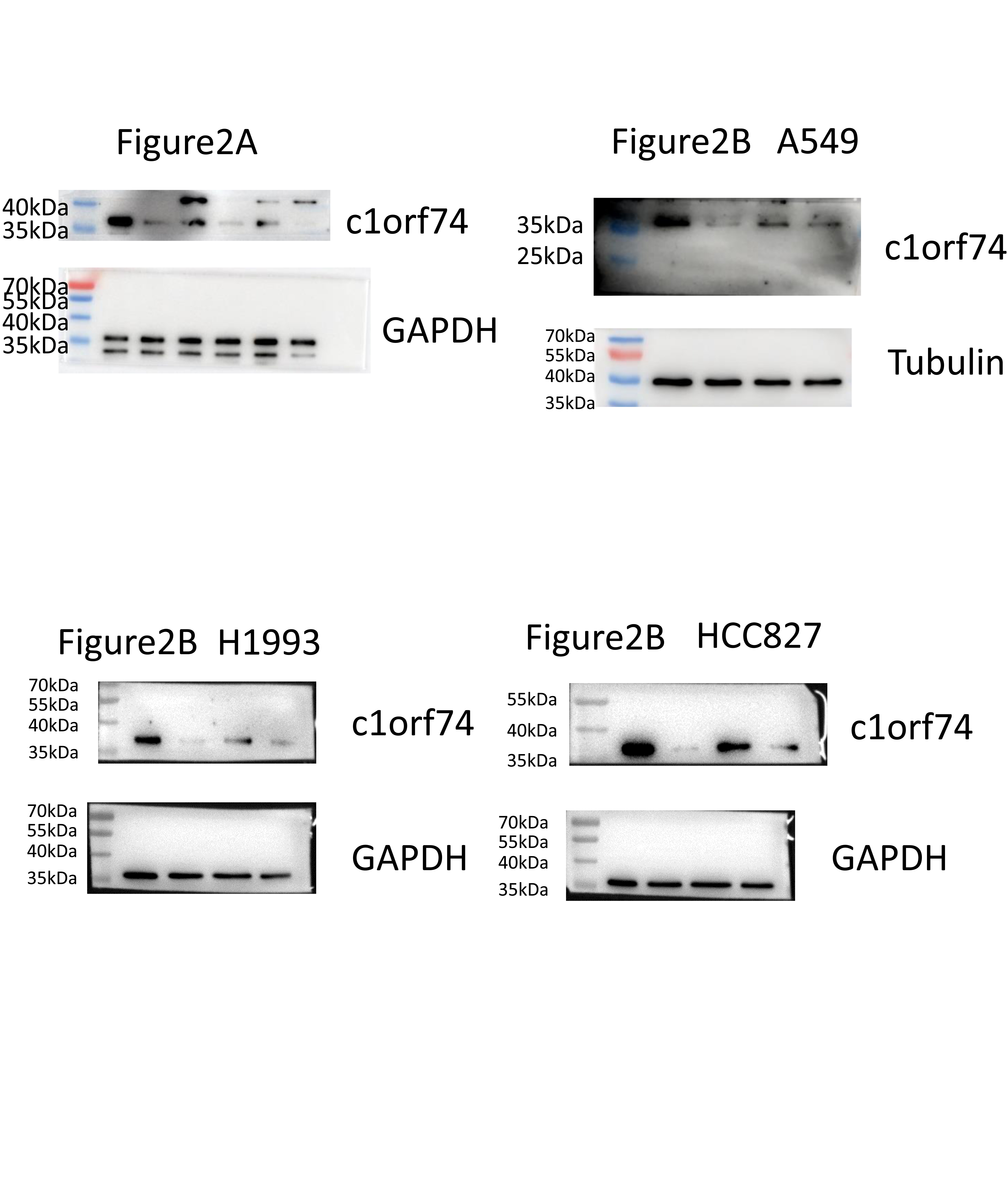

Supplement: Supplemental Information 1 [file peerj-10-13908-s001.zip › C1orf74 raw data/Fig 2A&B raw image.tif]

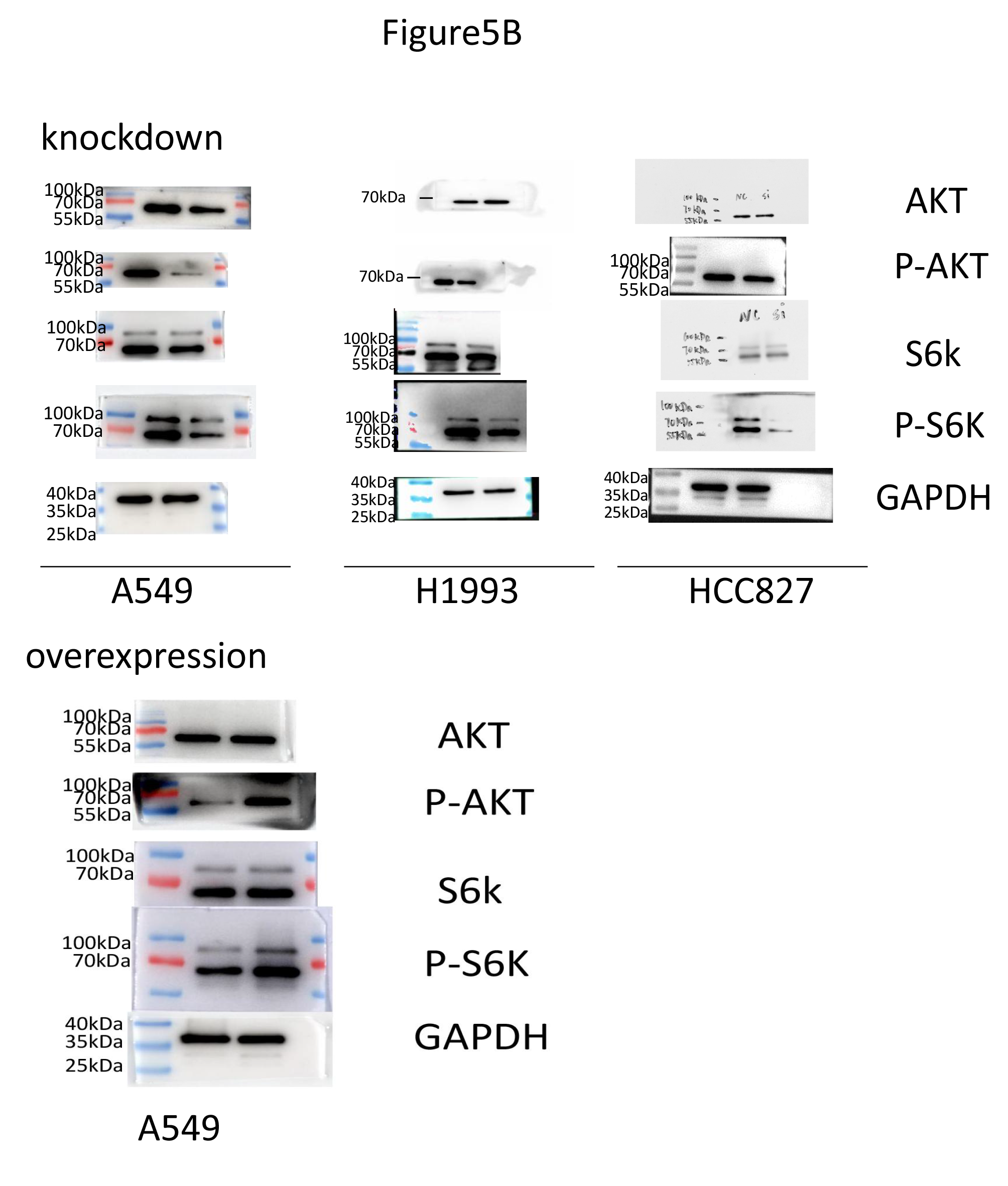

Supplement: Supplemental Information 1 [file peerj-10-13908-s001.zip › C1orf74 raw data/Fig 5B raw image.tif]

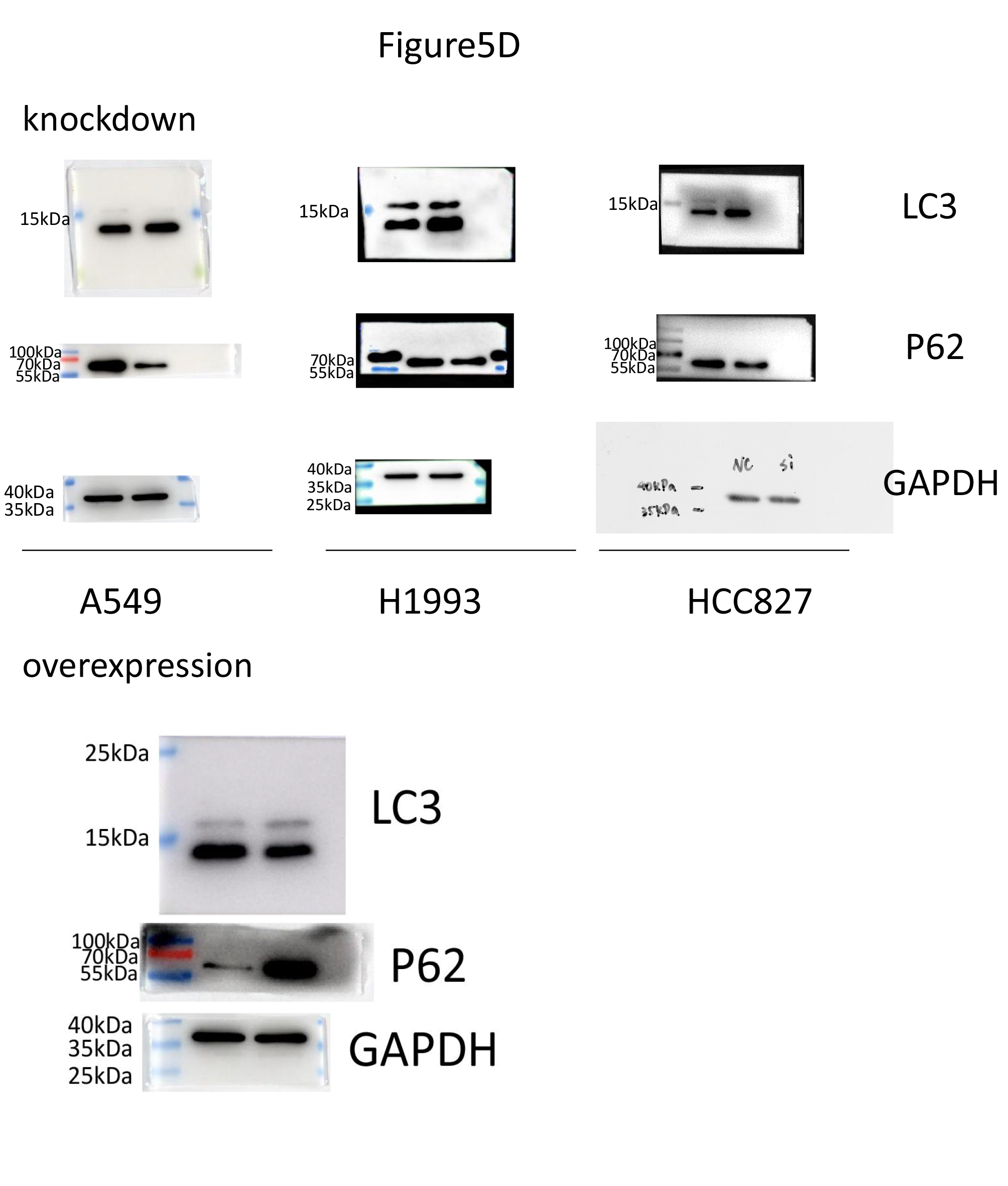

Supplement: Supplemental Information 1 [file peerj-10-13908-s001.zip › C1orf74 raw data/Fig 5D raw image.tif]

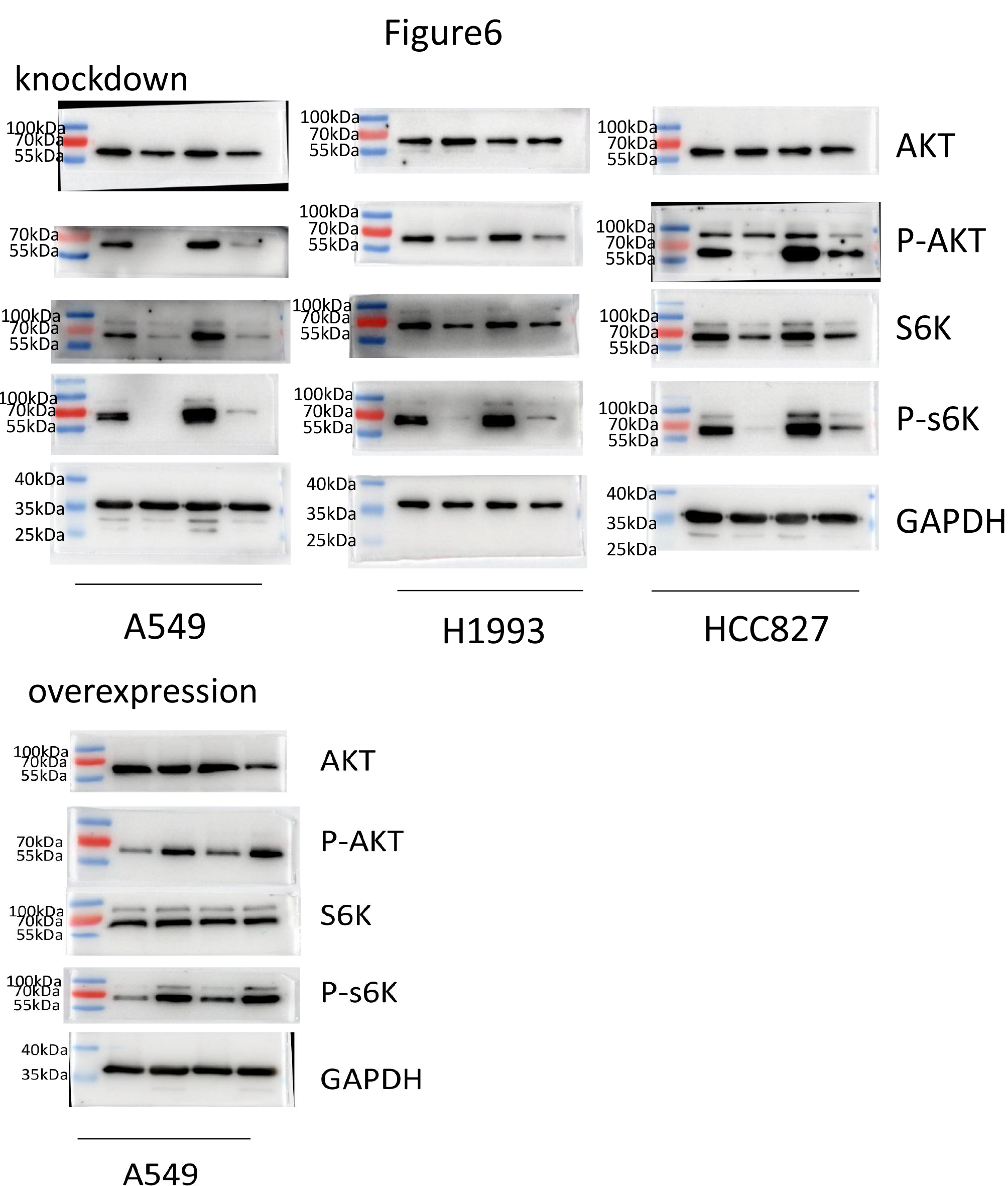

Supplement: Supplemental Information 1 [file peerj-10-13908-s001.zip › C1orf74 raw data/Fig 6 raw image.tif]
